# Spatiotemporal incidence of Zika and associated environmental drivers for the 2015-2016 epidemic in Colombia

**DOI:** 10.1038/sdata.2018.73

**Published:** 2018-04-24

**Authors:** Amir S. Siraj, Isabel Rodriguez-Barraquer, Christopher M. Barker, Natalia Tejedor-Garavito, Dennis Harding, Christopher Lorton, Dejan Lukacevic, Gene Oates, Guido Espana, Moritz U.G. Kraemer, Carrie Manore, Michael A. Johansson, Andrew J. Tatem, Robert C. Reiner, T. Alex Perkins

**Affiliations:** 1 Department of Biological Sciences and Eck Institute for Global Health, University of Notre Dame, 345 Galvin Hall, Notre Dame, IN 46556, USA; 2 Department of Epidemiology, Johns Hopkins Bloomberg School of Public Health, 615 N. Wolfe Street, Baltimore, MD 21205, USA; 3 Department of Pathology, Microbiology and Immunology, University of California, 5329 Vet Med 3A, Davis, CA 95616, USA; 4 WorldPop, Department of Geography and Environment, University of Southampton, University Road, Southampton, SO17 1BJ, UK; 5 Flowminder Foundation, Roslagsgatan 17, SE-11355, Stockholm, Sweden; 6 Institute for Disease Modeling, Bellevue, 3150 139th Ave SE, WA 98005, USA; 7 Spatial Ecology and Epidemiology Group, Department of Zoology, University of Oxford, South Parks Road, Oxford OX1 3PS, UK; 8 Harvard Medical School, 10 Shattuck Street, Boston, MA 02115, USA; 9 Boston Children’s Hospital, 300 Longwood Ave, Boston, MA 02115, USA; 10 Theoretical Biology and Biophysics Group, Los Alamos National Laboratory, Los Alamos, NM 87545, USA; 11 Centers for Disease Control and Prevention, 1324 Calle Canada, San Juan, PR 00920-3860, USA; 12 Center for Communicable Disease Dynamics, Harvard T.H. Chan School of Public Health, 677 Huntington Ave., Suite 506, Boston, MA 02115, USA; 13 Department of Global Health and Institute for Health Metrics and Evaluation, University of Washington, 2301 Fifth Ave., Suite 600, Seattle, WA 98121, USA

**Keywords:** Ecological epidemiology, Viral infection, Public health, Biogeography

## Abstract

Despite a long history of mosquito-borne virus epidemics in the Americas, the impact of the Zika virus (ZIKV) epidemic of 2015–2016 was unexpected. The need for scientifically informed decision-making is driving research to understand the emergence and spread of ZIKV. To support that research, we assembled a data set of key covariates for modeling ZIKV transmission dynamics in Colombia, where ZIKV transmission was widespread and the government made incidence data publically available. On a weekly basis between January 1, 2014 and October 1, 2016 at three administrative levels, we collated spatiotemporal Zika incidence data, nine environmental variables, and demographic data into a single downloadable database. These new datasets and those we identified, processed, and assembled at comparable spatial and temporal resolutions will save future researchers considerable time and effort in performing these data processing steps, enabling them to focus instead on extracting epidemiological insights from this important data set. Similar approaches could prove useful for filling data gaps to enable epidemiological analyses of future disease emergence events.

## Background & Summary

Zika virus (ZIKV) emerged as a pathogen of global concern in 2015 when it rapidly spread through the Americas and was associated with Guillain-Barré syndrome (GBS) in adults and congenital Zika syndrome (CZS) in fetuses and neonates^1^. Though ZIKV had been discovered several decades earlier, recognition of severe outcomes and the explosive nature of ZIKV epidemics was only established recently^2–5^. Moreover, an estimated 80% rate of asymptomatic infection^2,7,8^ and the presence of more infections with relatively mild symptoms who go unreported^9^ complicate efforts to estimate disease incidence and further make modeling the spread of ZIKV a challenging task. Despite these issues and the chronic lack of data at the appropriate spatio-temporal scales, efforts to understand the spatiotemporal dynamics of ZIKV rely heavily on access to data about its spatiotemporal drivers^6^.

ZIKV is transmitted primarily by *Aedes aegypti* mosquitoes, which also transmit chikungunya, yellow fever, and dengue viruses. Like these other viruses, ZIKV transmission is highly dependent on the environment. Climatic conditions, for example, regulate the population dynamics of vectors^10,11^, and the built environment plays an important role in human-vector interaction and in providing breeding grounds for mosquitoes^12^. Even though the importance of these factors is widely recognized, their specific roles are more difficult to understand but can be aided by model-based analysis combining epidemiological and environmental data^13^.

The availability of spatiotemporal incidence data is critical to both current and near-future responses and to planning for responses to emerging infectious disease outbreaks. For example, during the Ebola epidemic in 2014-2015, mathematical and statistical models using incidence data were critical to informing resource allocation and placement of new hospital beds^14^, plans for vaccine trials^15^, estimates of intervention effectiveness, and understanding how the outbreak started and where it spread in time and space^16^. Similarly, spatiotemporal ZIKV data has informed efforts to estimate the number of people at risk for infection and the number of pregnant women infected^6^. Such data are also potentially important for selecting sites for ZIKV vaccine trials^17^.

Despite the widely recognized importance of spatiotemporal incidence data, there is often limited availability of such data sets for emerging infectious diseases^18^. In the case of Zika, there has been some effort to broaden access to these data (e.g., the cdcepi Github repository^19^), but the data available through these settings are often not internally consistent and are not made available with important covariates, such as population and weather conditions. Colombia is one country for which data has been made available online by its Instituto Nacional de Salud^20^ and is of particular interest due to the high resolution of data there (available weekly for each of 1,122 municipalities). This data set is also of particular interest for modeling the spatio-temporal spread of ZIKV due to Colombia’s diverse landscape and because of substantial heterogeneity in the timing and intensity of ZIKV transmission there^21^. Together, these factors offer a unique opportunity to examine the role of environmental and social influences on the spread of ZIKV^22^.

In addition to spatiotemporal incidence data, several variables are commonly incorporated into analyses of the transmission dynamics of ZIKV and related pathogens^23,24^. First, temperature plays a dominant role in ZIKV transmission due to its influence on vector and virus life traits^25,26^. Because the effect of temperature on transmission depends not only on mean temperature but also on daily temperature range^27^, we include estimates of mean, minimum, and maximum daily temperature. Second, a number of metrics related to moisture—including precipitation, humidity, and normalized difference vegetation index (NDVI)—are commonly used for modeling mosquito population dynamics due to their relevance to the immature stages of the mosquito life cycle^11^. Third, we include spatiotemporal estimates of relative mosquito abundance^28^, a spatial estimate of purchasing power as a proxy for the effect of socioeconomic effects on mosquito-human contact^6,29^, and spatial estimates of travel time to allow for exploration of the effects of connectivity on spatiotemporal transmission dynamics^24^. Fourth, we include demographic projections^30^ of total population and annual births to allow for quantification of the population at risk of ZIKV infection and severe outcomes such as GBS and CZS.

Here, we collated data on the aforementioned variables at three administrative scales on a weekly basis between January 1, 2014 and October 1, 2016, which spans the majority of ZIKV transmission activity in Colombia. Our hope is that this effort will increase access to this data set and reduce duplication of the considerable effort required to process data for epidemiological analyses of ZIKV transmission dynamics.

## Methods

**Figure 1:**
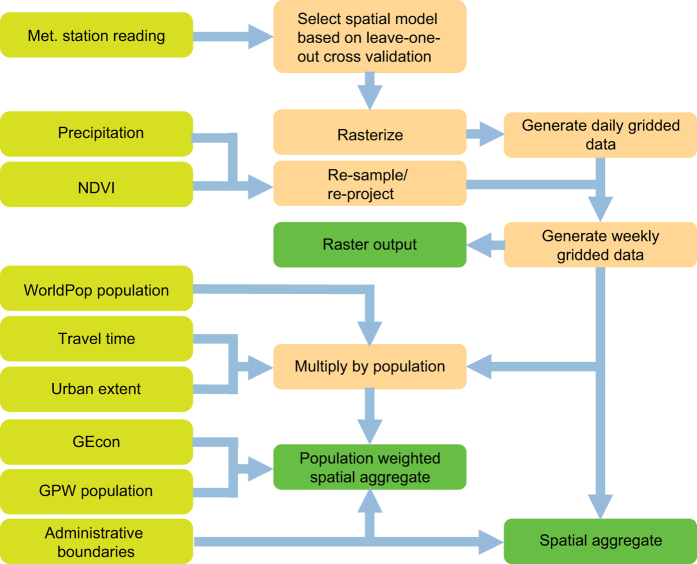
Schematic overview of the workflow used to produce the output raster files, and their spatial aggregates at the municipal, departmental, and national scales. The input stages are shown in yellow, and the processing stages are shown in orange, while the output stages are in green.

To achieve our central objective of assembling and collating multiple data sets pertaining to ZIKV transmission in Colombia, we first identified key data and then translated those data to comparable spatiotemporal resolution using a variety of methods. In some cases, this was as simple as downloading raster datasets and clipping them to shape files. In other cases, this involved statistical modelling to transform existing data products from certain scales into a single data product at some other desired scale. In all cases, our methods involved taking input data (Table 1) and generating output data (Table 2 (available online only), Data Citation 1) at a weekly timescale between January 1, 2014 and October 1, 2016 for each of three administrative scales (Fig. 1). Throughout, we generated output data at the national scale, for each of 33 departments, and for each of 1,122 municipalities, as defined by GIS shapefiles from the National Geographical Information System of Colombia^31^.

### Zika case reports

The weekly number of Zika cases, by municipality, was reconstructed using two data sources. The main data source was a website^20^ of the Colombian National Institute of Health (Instituto Nacional de Salud) where the official weekly reports on the cumulative number of Zika suspected and confirmed cases for each municipality have been published since the beginning of 2016.

While the peak of the Colombian epidemic occurred in 2016, a significant number of cases were reported during 2015. In order to capture this initial portion of the epidemic, we used an additional data source, also available in the INS website^20^. Unfortunately, the number of cases reported in the latter data source seemed to consistently underreport the total number of cases reported by the INS at the national scale. For example, while the official data source reports a cumulative number of 11,712 cases by the end of 2015, this secondary source only reports 3,875 cases for this same period. Therefore, in order to reconstruct the 2015 portion of the epidemic while accounting for the better known total number of cases, we multiplied the weekly 2015 data by a correction factor. This correction factor was calculated as the ratio between the cumulative number of cases reported by each municipality up to the first week of 2016 according to the official source and the alternative source. The raw and the corrected weekly counts for each municipality are included in the data set. To account for cases from unknown municipalities within a department, we also provide data at the departmental level.

### Human demographics

We obtained gridded population data across Colombia for the year 2015 at a resolution of 3 arc seconds (~93 m) from the WorldPop website (http://worldpop.org.uk). Similarly, we obtained high-resolution (30 arc seconds) unpublished gridded data on the number of births for the year 2015 from the WorldPop project. These high-resolution products were developed to ensure consistencies with subnational data on sex and age structures, as well as subnational age-specific fertility rates, while adjustments on births were made at subnational scales using data from the government of Colombia^32,33^, followed by national-level adjustments to contemporary numbers based on 2012 and 2015 United Nations Population Division data^30,34^.

### Spatial aggregation of covariates

**Figure 2:**
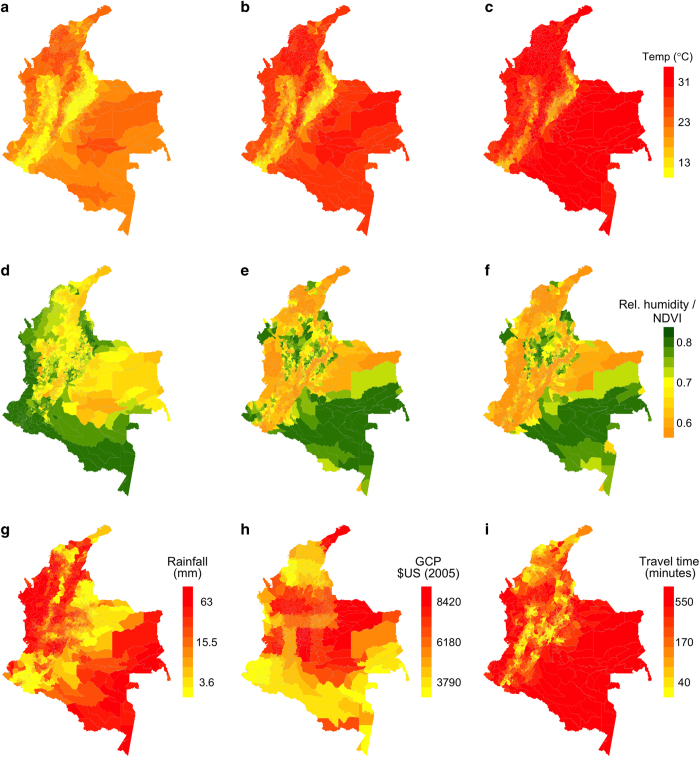
Illustrative maps of municipality level weighted output variables for a single sample week. Variables include minimum temperature (**a**), mean temperature (**b**) maximum temperature (**c**), relative humidity (**d**), NDVI from Terra MODIS (**e**) NDVI from Aqua MODIS (f), total rainfall (**g**) average per capita gross cell product in 2005 US$ standard value (**h**) and average travel time to major cities in 2000 (i).

**Figure 3:**
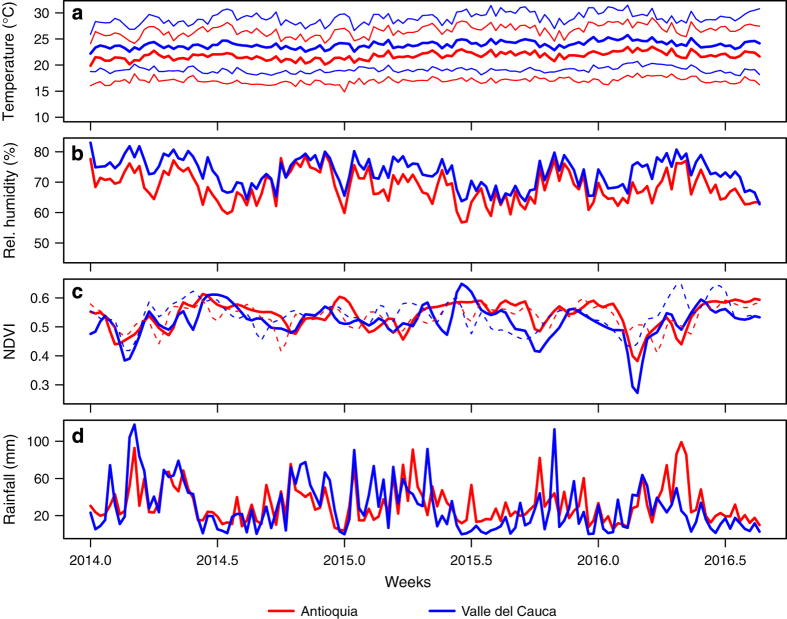
Illustration of weekly time-series outputs aggregated at the departmental scale for the departments of Antioquia (red) and Valle del Cauca (blue) for the period January 1, 2014 to October 1, 2016. Time-series shown are for mean temperature (bold lines), minimum temperature (lower lines) and maximum temperature (upper lines) (**a**), relative humidity (**b**), NDVI from Terra (solid lines) and Aqua (dashed lines) MODIS (**c**), and precipitation (**d**).

Aggregation of raster data at the level of administrative units requires some assumption about how raster values should be weighted to obtain a single value for an administrative unit. Due to the fact that Zika virus transmission occurs predominantly in human-dominated areas, we used human population (WorldPop Project) as our weighting variable. We applied this weighting procedure to aggregate all covariates at municipal (e.g., as in Fig. 2), departmental (e.g., as in Fig. 3), and national levels.

### Aedes aegypti abundance

We obtained one hundred posterior samples of *Aedes aegypti* occurrence probabilities in raster format, from the published work of Kraemer *et al.*^28^, which we used to derive weekly mosquito abundance measures for all 52 weeks of the year. We based our method on the assumption that *m* mosquitoes at time *t*, *m*(*t*), can be represented by a Poisson distribution with rate parameter *λ*=−ln(l-*occurrence probability*), consistent with existing ZIKV transmission models^29,35^. We obtained such an estimate of the relative density of mosquitoes across a 4.65 km x 4.65 km grid for each of 52 weeks. In addition, we generated aggregated values at the municipality, department and national scales after weighting the raster data values by population (see the section on Spatial aggregation of covariates).

### Temperature

We downloaded meteorological readings from 30 stations across continental Colombia from National Oceanic and Atmospheric Administration (NOAA)’s Climate Data Online, an online archive of daily meteorological readings^36^. The variables we extracted from this data set included minimum daily temperature, maximum daily temperature, mean daily temperature, and relative humidity, all on a daily basis between January 1, 2014 and October 1, 2016.

To facilitate interpolation of these climate variables across a more complete spatial coverage of the country, we downloaded a digital elevation dataset at a resolution of 30 arc seconds from the Global 30 Arc-Second Elevation (GTOPO30) product^37^. Similarly, we downloaded the WorldClim gridded long-term average of monthly minimum temperature, maximum temperature, and precipitation at a 4.65 km x 4.65 km spatial resolution^38^, as well as NOAA’s Climate Prediction Center (CPC) global monthly mean air temperature at 0.5 arc-degrees resolution^39^.

To generate smooth, high-resolution surfaces of climate variables based on calibration to point readings from the 30 meteorological stations, we tested two approaches of spatial interpolation: (a) using non-parametric surface fitting with thin plate splines (TPS) with or without fixed-factor covariates^40^; (b) using spatial models (kriging) with or without covariates^41^. We selected the best interpolation models for each environmental variable based on leave-one-out cross validation, as described in the Technical Validation section.

The thin plate spline (TPS) follows the general form,

(1)Y(x)=µ(x)+P(x)+ε

where *Y* is the dependent variable evaluated at location *x*, *μ* is the fixed effect component of the model with optional covariates at location *x*_*i*_, *P* is the implicit spline polynomial function over the spatial coordinates, and *ε* is measurement error, assumed to be uncorrelated across sites and normally distributed with mean zero and standard deviation *σ*.

The kriging approach follows the concept that spatial autocorrelation is dependent on distance between locations. We used the krige function in the geoR library of R with parameters chosen based on maximum-likelihood estimation^42^. The model of a spatial process indexed by spatial locations *x*_*i*_ follows

(2)Y(x)=µ(x)+S(x)+ε

where *Y* is the dependent variable evaluated at location *x*, *μ* is the fixed effect component of the model at location *x*_*i*_, *S* is a stationary Gaussian process with variance *σ*^2^ (partial sill) and a correlation function parametrized by *φ* (range), and *ε* is the error term with its variance *τ*^2^ (nugget variance). When *μ* is included, the trend is implemented using lm, the regression model function in R, and *S*(*x*) is fitted to the residuals of the regression model^41^.

Due to Colombia’s proximity to the Equator, we ignored the small effect of distance distortion arising from non-projected spatial layers on both models^43^. Because our goal is generating daily surfaces of climate variables, rather than developing a predictive model that works for days outside those to which we fitted the model, we treated every day separately and fitted a model for each day between January 1, 2014 and October 1, 2016 for which data was available. In addition to generating daily raster outputs and aggregating them at weekly time steps, we generated aggregated values at the municipality (Figs 2a–c), department (Fig. 3a) and national scales after weighting the raster data values by population (see the section on Spatial aggregation of covariates).

### Relative humidity

Rather than interpolating relative humidity directly based on station readings (which showed poor estimates in preliminary results), we approached the task of estimating relative humidity indirectly. First, we spatially interpolated weather station measurements of mean dew point temperature from the 30 stations across Colombia. This was followed by calculating relative humidity across the 4.65 km x 4.65 km grid based on interpolated mean temperature and dew point temperature, using the August-Roche-Magnus approximation for the saturation vapour pressure of water in air^44^, which follows

(3)RH=exp(−ab(T−Td)(b+Td)(b+T))

where *T* and *T*_*d*_ are the mean temperature and dew point temperature in °C and *a*=17.271 and *b*=237.7 °C^44^. Finally, in addition to generating daily raster outputs and aggregating them at weekly time steps, we generated aggregated values at the municipality (Fig. 2d), department (Fig. 3b) and national scales after weighting the raster data values by population (see the section on Spatial aggregation of covariates).

### Normalized Difference Vegetation Index (NDVI)

Satellite-based technologies have been used to capture spatial variation in environmental factors related to vector population dynamics^45–47^, including a commonly used index called Normalized Difference Vegetation Index (NDVI) that captures the vegetation cover of regions. To account for spatial and temporal variation in vegetation cover that could influence habitat suitability for *Ae. aegypti*, the primary ZIKV vector, we downloaded NASA’s Moderate Resolution Image Spectro-radiometer (MODIS-Terra and Aqua version 13A2) vegetation indices at 16-day temporal and 1 km x 1 km spatial resolutions (Data Citation 2, Data Citation 3). These products have similar sensors but differ in their orbits as well as their daily hours and directions of crossing the equator. We linearly interpolated between data points (days on which data was reported) to generate a daily time series before aggregating the data back to a weekly resolution. In addition, we generated aggregated values at the municipality (Figs 2e and f), department (Fig. 3c) and national scales after weighting the raster data values by population (see the section on Spatial aggregation of covariates).

### Precipitation

Among the climate datasets we explored, precipitation proved to be the most spatially variable, making it difficult to rely on spatial models to make accurate estimates. Our attempt of spatial interpolation of precipitation using ordinary kriging resulted in large deviations from the observed values of the 30 stations obtained from NOAA. As an alternative, we used satellite-based data from NOAA’s Center for Satellite Applications and Research (STAR). We downloaded daily layers of the STAR rainfall estimates at ~4 km x 4 km resolution^48^. Once we download the daily products, we subset and resampled them into our standard resolution (4.65 km x 4.65 km) and spatial extent compatible with the other variables considered, before averaging across each consecutive seven days to generate weekly gridded data. In addition, we generated aggregated values at the municipality (Fig. 2g), department (Fig. 3d) and national scales after weighting the raster data values by population (see the section on Spatial aggregation of covariates).

### Geographically based Economic data (G-Econ)

To account for socioeconomic differences, which are potentially associated with contact between humans and the vector, we used one-degree resolution gridded estimates of 2005 purchasing power parity (PPP) adjusted gross domestic product (GDP)^49^. To express the values in per capita, we divided the gridded GDP by the corresponding population, the latter obtained from the Gridded Population of the World product (v3)^50^ after resampling the latter to one-degree resolution. We chose this version of gridded population data for this task given that it was the one originally used to generate the 2005 gridded GDP values. Cells with missing values were imputed with the mean of the surrounding eight grid cell values. Once we obtained a complete grid layer at a resolution of one-degree (~111 km at the equator), we resampled the layer, without smoothing, to a resolution of 4.65 km x 4.65 km to match the resolution of all other gridded layers. We additionally computed aggregated results at the municipality, department and national levels after weighting them by the distribution of population (in the year 2005) within each administrative unit (see the section on Spatial aggregation of covariates).

### Travel time

To account for the general accessibility of each municipality and department, we used travel time data downloaded from the European Commission’s Joint Research Center at a resolution of 30 arc seconds^51^. This definition of travel time is a measure of overall accessibility rather than of frequency of travel. It is defined as the average length of time (in minutes) it takes individuals in a region to travel to the nearest location with a population greater than 50,000. Large travel time is indicative of a region whose population lives relatively far from urban centers. This gridded dataset has minutes of land-based travel time to the nearest settlement with population greater than 50,000 (as of the year 2000). The data is developed using a cost-distance model, which accounts for travel time increments based on the available transport networks and other environmental and political factors^51^. We aggregated travel time weighted by population at the municipal level to generate estimates of travel time for each municipality and similarly for each department (see the section on Spatial aggregation of covariates).

### Urban population

To identify the level of urbanization in each grid cell, we downloaded the MODIS global 2002 urban extent raster dataset^52,53^, which has a binary (0 or 1) value for each 500 m x 500 m grid cell around the globe. By counting the number of high-resolution urban grid cells that fall within each standard grid cell of 4.65 km x 4.65 km, we were able to generate a gridded product of percentage of the physical grid cell that is urban. Furthermore, in combination with the population raster we obtained from WorldPop^30^, we were able to generate a gridded estimate of urban population at each 500 m x 500 m grid cell in Colombia.

### Code availability

The code used to generate all gridded datasets and aggregating at municipal, departmental, and national levels is freely available for download from GitHub at https://github.com/asiraj-nd/zika-colombia^54^. This code utilizes the R programming language^42^ and Python version 2.7.10. Further explanation of the code is provided in a readme file in the repository on GitHub^54^.

## Data Records

All output datasets described in this article (Data Citation 1) are publicly and freely available through Dryad Digital Repository. The datasets stored in the datadryad.org Repository represent the ones produced at the time of writing, and will be preserved in their published form. Datasets of interest can be obtained by downloading the corresponding zipped archive files (Table 2 (available online only)).

## Technical Validation

Most datasets obtained from other sources have already been validated by independent studies^30,38,39,48–53^. We therefore limited our validation to the interpolated climate model outputs developed here by comparing spatial interpolation results to data from the 30 meteorological stations across Colombia. These comparisons were made for the two modeling approaches and for different combinations of covariates for each outcome: mean temperature, maximum temperature, minimum temperature, precipitation, and relative humidity.

We used three metrics to compare model performance: mean absolute error, coefficient of variation, and Pearson’s correlation coefficient (COR). Mean absolute error (MAE) is the mean absolute difference between predictions and observations over *n* data points:

(4)MAE=1n∑i=1n|yˆi−yi|

We also used relative MAE (of two models), which is the ratio of the two MAEs. A relative MAE *m* of models A and B respectively, would indicate that predictions from model A were (1-*m*)% closer to the observed values than those from model B for an *m* value less than 1. The coefficient of variation (CV) evaluates the extent to which large values are dispersed relative to their mean value. It is the ratio of the root mean square error (RMSE) to the mean of observed values,

(5)CV=1y¯1n∑i=1n(yˆi−yi)2

Results of our comparison are described in Table 3. Overall, the ordinary kriging approach had higher accuracy for temperature (mean, maximum, and minimum) and relative humidity based on all three metrics. Model results also revealed that using other covariates, such as altitude and secondary climate data, improved interpolation results for temperature and relative humidity.

## Usage Notes

This compilation of datasets can facilitate a variety of studies relevant to vector-borne disease epidemiology in Colombia. The archive provides ready to use data both in a raster format with resolution of 5km x 5km, and at administrative units of municipal, departmental, and national scales.

These datasets have several limitations. First, the 30 meteorological stations used in generating climate surfaces are sparsely and unevenly distributed over Colombia, leading to uncertainty in the outputs. Moreover, some of the original gridded data we obtained had differing resolutions, including 0.1 arc-degrees (GPM), 0.5 arc-degrees (CPC), and 1 arc-degree (G-Econ). This meant that we had to resample these gridded products (GPM, CPC, GEcon) with crude estimates based on average values over a large swath of grid cells. Further, unlike all other products we used that were non-projected geographic WGS1984 raster files, the Tera and Aqua MODIS NDVI products were in sinusoidal projections, causing some distortions when re-projected to match population layers used in weighting.

In addition to spatial discrepancies, we also had to overcome the relatively poor temporal resolutions of Tera and Aqua MODIS NDVI products (which come at 16-day intervals) by linearly interpolating between two data points to fill in the 15 days in between, before aggregating the results at weekly time steps. Furthermore, daily satellite based rainfall data from NOAA assume 12:00-12:00 hour-day, which could potentially cause slight inconsistencies, despite the data finally being aggregated at weekly time steps. Other limitations include the modifiable area unit problem, which arises from disparities in the arbitrary sizes and borders of the administrative units which may bias aggregations based on these borders.

## Additional information

**How to cite this article:** Siraj, A. S. *et al.* Spatiotemporal incidence of Zika and associated environmental drivers for the 2015-2016 epidemic in Colombia. *Sci. Data* 5:180073 doi: 10.1038/sdata.2018.73 (2018).

**Publisher’s note:** Springer Nature remains neutral with regard to jurisdictional claims in published maps and institutional affiliations.

## Supplementary Material



## Figures and Tables

**Table 1:** Input datasets, used to generate gridded and administrative aggregate outputs.

**Name**	**Acquisition year**	**Source**	**Version, Publication year, License**	**Data Type**	**Spatial Resolution**	**Format/ Pixel Type & Depth**	**Spatial Reference**	**Spatial Coverage**
GTOPO30 Gridded Elevation	<1996	USGS^37^	1996, CC0 1.0	Elevation, continuous raster	30” (~930 m)	Geo-tiff/flt32	GCS WGS 1984	Regional
CPC Surface Air temperature	2014–2016	Fan & van den Dool^39^	2008, CC0 1.0	Monthly surface air temperature, continuous raster	1800” (~56 km)	ESRI grids/flt32	GCS WGS 1984	Global
Worldclim Average Temperature	1960–1990	Hijmans R.J., *et al.*^38^	v1, 2005, CCBY 4.0	Average monthly temperature, continuous raster	150” (~4.65 km)	Geo-tiff/flt32	GCS WGS 1984	Global
Worldclim Minimum Temperature	1960–1990	Hijmans R.J., *et al.*^38^	v1, 2005, CCBY 4.0	Average monthly minimum temperature, continuous raster	150” (~4.65 km)	Geo-tiff/flt32	GCS WGS 1984	Global
Worldclim Maximum Temperature	1960-1990	Hijmans R.J., *et al.*^38^	v1, 2005, CCBY 4.0	Average monthly maximum temperature, continuous raster	150” (~4.65 km)	Geo-tiff/flt32	GCS WGS 1984	Global
Daily Station Mean Temperature	2014–2016	NOAA^36^	2016, CC0 1.0	Daily mean temperature reading from 30 stations, continuous vector	Comparable to 1” (~30 m)	HTML/flt32	GCS WGS 1984	Colombia
Daily Station Minimum temperature	2014–2016	NOAA^36^	2016, CC0 1.0	Daily minimum temperature reading from 30 stations, continuous vector	Comparable to 1” (~30 m)	HTML/flt32	GCS WGS 1984	Colombia
Daily Station Maximum Temperature	2014–2016	NOAA^36^	2016, CC0 1.0	Daily maximum temperature reading from 30 stations, continuous vector	Comparable to 1” (~30 m)	HTML/flt32	GCS WGS 1984	Colombia
Daily Station Relative Humidity	2014–2016	NOAA^36^	2016, CC0 1.0	Daily relative humidity reading from 30 stations, continuous vector	Comparable to 1” (~30 m)	HTML/flt32	GCS WGS 1984	Colombia
Daily Mean Dew Point Temperature	2014–2016	NOAA^36^	2016, CC0 1.0	Daily mean dew point temperature reading from 30 stations, continuous vector	Comparable to 1” (~30 m)	HTML/flt32	GCS WGS 1984	Colombia
Gridded Population of the World (GPW)	2005	CIESIN^50^	v3, 2004, CCBY 4.0	Global Population Estimates, continuous raster	150” (~4.65 km)	Geo-tiff/flt32	GCS WGS 1984	Global
Confirmed and Suspected Cumulative ZIKV Cases	2015–2016	INS^20^	2016	Weekly suspected and confirmed cumulative ZIKV cases by municipality from two INS sources	NA	CSV/flt32	NA	Colombia
Occurrence Probability of *Aedes aegypti*	1960–2014	Kraemer *et al.* 2015^28^	2015, Author	Global occurrence probabilities of *Aedes aegypti*, continuous raster	150” (~4.65 km)	Geo-tiff/flt32	GCS WGS 1984	Global
GEcon – Gross Cell Product	2005	Nordhaus^49^	2006, CCBY 4.0	Global gridded gross cell product, continuous raster	3600” (~111 km)	XLS/flt32	GCS WGS 1984	Global
WorldPop Population	2015	WorldPop^30^	2016, CCBY 4.0	Population count, continuous raster	3” (~93 m)	Geo-tiff/flt32	GCS WGS 1984	Colombia
WorldPop Births	2015	WorldPop^30^	2016, Author	Count of births, continuous raster	30” (~93 m)	Geo-tiff/flt32	GCS WGS 1984	Colombia
MODIS –MOD13A2 NDVI	2014–2016	Didan K. (Data Citation 2)	v6, 2015, CC0 1.0	16-day NDVI from Terra MODIS, continuous raster	30” (~930 m)	HDF-EOS tiles/uint8	Sinusoidal	Global
MODIS –MYD13A2 NDVI	2014–2016	Didan K (Data Citation 3)	v6, 2015, CC0 1.0	16-day NDVI from Aqua MODIS, continuous raster	30” (~930 m)	HDF-EOS tiles/uint8	Sinusoidal	Global
NOAA’s Satellite Applications and Research Rainfall Estimates	2015–2016	NOAA^44^	2016, CC0 1.0	Daily precipitation estimates from satellites, continuous raster	360” (~11 km)	Net-CDF /uint8	GCS WGS 1984	Global
Travel Time to Major Cities	2000	Nelson A.^51^	2008, CCBY 3.0	Travel time, continuous raster	30” (~930 m)	Flt/flt32/flt32	GCS WGS 1984	Global
MODIS 500m Global Urban Extent	2002	Schneider et al.^52,53^	2009, CCBY 3.0	Urban extent	15” (~465 m)	Flt/flt32/flt32	GCS WGS 1984	Global
Administrative Boundaries of Colombia	2015	SIGOT, Colombia^31^	2015	Municipal administrative boundaries, vector	Comparable to 15” (~465 m)	ESRI polygon shapefile tiles	GCS WGS 1984	Colombia

**Table 2:** Output datasets, compiled and generated at different spatial scales

**Name**	**Acquisition year**	**Source**	**Publication year**	**Data Type**	**Spatial Resolution**	**Format/ Pixel Type & Depth**	**Spatial Reference**	**Spatial Coverage**
Mean Temperature Time Series	2014–2016	Model derived based on GTOPO30 elevation, WorldClim, and Daily Station data			Weekly mean temperature, continuous raster	150” (~4.65 km)	Raster brick/flt32	GCS WGS 1984	Colombia
Minimum Temperature Time Series	2014–2016	Model derived based on GTOPO30 elevation, Mean Temperature Time Series and Daily Station data			Weekly minimum temperature, continuous raster	150” (~4.65 km)	Raster brick/flt32	GCS WGS 1984	Colombia
Maximum Temperature Time Series	2014–2016	Model derived based on GTOPO30 elevation, WorldClim and Daily Station data			Weekly maximum temperature, continuous raster	150” (~4.65 km)	Raster brick/ flt32	GCS WGS 1984	Colombia
Precipitation Time Series	2014–2016	Aggregated from NOAA’s Satellite Applications and Research Rainfall Estimates			Weekly mean relative humidity, continuous raster	150” (~4.65 km)	Raster brick/flt32	GCS WGS 1984	Colombia
Mean Relative Humidity Time Series	2014–2016	Model derived based on GTOPO30 elevation, Mean Temperature Time Series and Daily Station data			Weekly mean relative humidity, continuous raster	150” (~4.65 km)	Raster brick/flt32	GCS WGS 1984	Colombia
NDVI –Terra MODIS Time Series	2014–2016	Temporally interpolated from MODIS –MOD13A2 NDVI			Weekly mean NDVI from Terra MODIS, continuous raster	150” (~4.65 km)	Raster brick/flt32	Sinusoidal	Colombia
NDVI –Aqua MODIS Time Series	2014–2016	Temporally interpolated from MODIS –MYD13A2 NDVI			Weekly mean NDVI from Aqua MODIS, continuous raster	150” (~4.65 km)	Raster brick/flt32	Sinusoidal	Colombia
Gridded *Aedes aegypti* Abundance	Derived from Occurrence Probability of *Aedes aegypti*			*Aedes aegypti* abundance for each week of the year, continuous raster	150” (~4.65 km)	Raster brick/flt32	GCS WGS 1984	Colombia	
Gridded per-capita gross cell product	2005	Derived from GEcon and Gridded Population of the World			Per-capita gross cell product, continuous raster	150” (~4.65 km)	BIL/flt32	GCS WGS 1984	Colombia
Travel Time to Major Cities	2000	Nelson A.^51^	2008	Travel time, continuous raster	30” (~930 m)	BIL/flt32	GCS WGS 1984	Colombia	
WorldPop Population	2015	WorldPop^30^	2016	Population count, continuous raster	3” (~93 m)	BIL/flt32	GCS WGS 1984	Colombia	
WorldPop Births	2015	WorldPop^30^	2016	Count of births, continuous raster	3” (~93 m)	BIL/flt32	GCS WGS 1984	Colombia	
Urban Population	2015	Derived from MODIS 500 m Global Urban Extent and WorldPop Population			Population residing in urban areas	15” (~465 m)	BIL/flt32	GCS WGS 1984	Colombia
Administrative Boundaries of Colombia	2015	IGAC, Colombia^31^	2015	Municipal administrative boundaries, vector	Comparable to 15” (~465 m)	ESRI polygon shapefile tiles	GCS WGS 1984	Colombia	
Confirmed and Suspected ZIKV Cases	Derived from Confirmed and Suspected Cumulative ZIKV Cases			Weekly suspected and confirmed ZIKV cases by administrative unit, table	NA	CSV/flt32	NA	Municipality, Department, National	
Per-capita gross cell product Aggregate	Derived from Gridded per-capita gross cell product and Administrative Boundaries of Colombia			Mean Per-capita gross cell product, by administrative unit, table	NA	CSV/flt32	NA	Municipality, Department, National	
Population Aggregate	Derived from WorldPop Population and Administrative Boundaries of Colombia			Population by administrative units, table	NA	CSV/flt32	NA	Municipality, Department, National	
Births Aggregate	Derived from WorldPop births and Administrative Boundaries of Colombia			Births by administrative units, table	NA	CSV/flt32	NA	Municipality, Department, National	
Urban Population Aggregate	Derived from WorldPop Population and MODIS 500m Global Urban Extent			Urban population by administrative unit, table	NA	CSV/flt32	NA	Municipality, Department, National	
Population Weighted Aedes aegypti Abundance Aggregate	Derived from Gridded *Aedes aegypti* Abundance, WorldPop Population and Administrative Boundaries of Colombia			Population weighted *Aedes aegypti* abundance for each week of the year by administrative unit, table	NA	CSV/flt32	NA	Municipality, Department, National	
Population Weighted Travel Time to Major Cities	Derived from Travel Time to Major Cities, WorldPop Population and Administrative Boundaries of Colombia			Travel time to major cities weighted by population, table	NA	CSV/flt32	NA	Municipality, Department, National	
Population Weighted Mean Temperature Time Series	Derived from Mean Temperature Time Series, WorldPop Population and Administrative Boundaries of Colombia			Weekly mean temperature weighted by population, table	NA	CSV/flt32	NA	Municipality, Department, National	
Population Weighted Minimum Temperature Time Series	Derived from Minimum Temperature Time Series, WorldPop Population, and Administrative Boundaries of Colombia			Weekly minimum temperature weighted by population, table	NA	CSV/flt32	NA	Municipality, Department, National	
Population Weighted Maximum Temperature Time Series	Derived from Maximum Temperature Time Series, WorldPop Population, and Administrative Boundaries of Colombia			Weekly maximum temperature weighted by population, table	NA	CSV/flt32	NA	Municipality, Department, National	
Population Weighted Precipitation Time Series	Derived from Precipitation Time Series, WorldPop Population, and Administrative Boundaries of Colombia			Weekly total precipitation weighted by population, table	NA	CSV/flt32	NA	Municipality, Department, National	
Population Weighted Relative Humidity Time Series	Derived from Mean Relative Humidity Time Series, WorldPop Population, and Administrative Boundaries of Colombia			Weekly mean relative humidity weighted by population, table	NA	CSV/flt32	NA	Municipality, Department, National	
Population weighted NDVI – Terra MODIS	Derived from NDVI –Terra MODIS Time Series, WorldPop Population, and Administrative Boundaries of Colombia			Weekly mean NDVI from MODIS Terra weighted by population, table	NA	CSV/flt32	NA	Municipality, Department, National	
Population weighted NDVI – Aqua MODIS	Derived from NDVI –Aqua MODIS Time Series, WorldPop Population, and Administrative Boundaries of Colombia			Weekly mean NDVI from MODIS Aqua weighted by population, table	NA	CSV/flt32	NA	Municipality, Department, National	

**Table 3:** Comparisons of model validation results for mean temperature, minimum temperature, maximum temperature and relative humidity based on leave-one-out approach.

**Spatial interpolation method**	**Response variable and fixed factors used**	**MAE**	**CV**	**COR**
**Mean temperature**				
Thin Plate Spline	None	3.85	0.21	0.38
	Altitude	1.67	0.09	0.90
	Altitude, distance to ocean	1.75	0.1	0.88
	Altitude, CPC temp	3.58	0.19	0.66
	Altitude, Worldclim temp	1.21	0.07	0.95
Ordinary kriging	Altitude, Worldclim temp, CPC temp	1.23	0.07	0.94
	None	3.45	0.21	0.43
	Altitude, Worldclim temp	1.09	0.06	0.96
	Altitude, Worldclim temp, CPC temp	1.13	0.06	0.95
**Minimum temperature**				
Ordinary kriging	Altitude, Worldclim temp	1.26	0.08	0.95
	Altitude, interpolated mean temp.	1.13	0.07	0.96
	Altitude, Worldclim temp, interpolated mean temp.	1.46	0.1	0.93
**Maximum temperature**				
Ordinary kriging	Altitude, Worldclim temp	1.54	0.07	0.92
	Altitude, interpolated mean temp.	2.02	0.1	0.85
	Altitude, Worldclim temp, interpolated mean temp.	2.03	0.1	0.85
**Relative humidity**^a^				
Ordinary kriging	Altitude, Worldclim temp	5.49	0.3	0.86
	Altitude, interpolated mean temp.	1.40	0.1	0.92
	Altitude, Worldclim temp, interpolated mean temp.	1.46	0.1	0.91
Larger MAE and CV values indicate worse fits, while larger COR values indicate better fit.				

^a^Derived using Equation 3.
